# Active Surveillance of Asymptomatic, Presymptomatic, and Oligosymptomatic SARS-CoV-2-Infected Individuals in Communities Inhabiting Closed or Semi-closed Institutions

**DOI:** 10.3389/fmed.2021.640688

**Published:** 2021-02-04

**Authors:** Nicolás Ambrosis, Pablo Martin Aispuro, Keila Belhart, Daniela Bottero, Renée Leonor Crisp, María Virginia Dansey, Magali Gabrielli, Oscar Filevich, Valeria Genoud, Alejandra Giordano, Min Chih Lin, Anibal Lodeiro, Felipe Marceca, Nicolás Pregi, Federico Remes Lenicov, Luciana Rocha-Viegas, Erika Rudi, Guillermo Solovey, Eugenia Zurita, Adali Pecci, Roberto Etchenique, Daniela Hozbor

**Affiliations:** ^1^Laboratorio VacSal, Facultad de Ciencias Exactas, Instituto de Biotecnología y Biología Molecular, Universidad Nacional de La Plata y CCT La Plata-CONICET, La Plata, Argentina; ^2^Universidad de Buenos Aires, Facultad de Ciencias Exactas y Naturales, Departamento de Química Biológica, Buenos Aires, Argentina; ^3^Universidad de Buenos Aires, Facultad de Ciencias Exactas y Naturales, Unidad de Microanálisis y Métodos Físicos en Química Orgánica, CONICET, Buenos Aires, Argentina; ^4^Escuela de Ciencia y Tecnología de la Universidad Nacional de San Martín, CONICET, Buenos Aires, Argentina; ^5^Universidad de Buenos Aires, Facultad de Ciencias Exactas y Naturales, Instituto del Cálculo, CONICET, Buenos Aires, Argentina; ^6^Laboratorio de Genética, Facultad de Ciencias Agrarias y Forestales, Universidad Nacional de La Plata, La Plata, Argentina; ^7^Universidad de Buenos Aires, Facultad de Ciencias Exactas y Naturales, Departamento de Matemática, Instituto de Investigaciones Matemáticas “Luis Santaló”, CONICET, Buenos Aires, Argentina; ^8^Universidad de Buenos Aires, Facultad de Ciencias Exactas y Naturales, Instituto de Química Biológica de la Facultad de Ciencias Exactas y Naturales, CONICET, Buenos Aires, Argentina; ^9^Universidad de Buenos Aires, Instituto de Investigaciones Biomédicas en Retrovirus y SIDA, CONICET, Buenos Aires, Argentina; ^10^Universidad de Buenos Aires, Facultad de Ciencias Exactas y Naturales, Instituto de Fisiología, Biología Molecular y Neurociencias, CONICET, Buenos Aires, Argentina; ^11^Universidad de Buenos Aires, Facultad de Ciencias Exactas y Naturales, Departamento de Química Inorgánica, Analítica y Química Física, Instituto de Química Física de los Materiales, Medio Ambiente y Energía, CONICET, Buenos Aires, Argentina

**Keywords:** SARS-CoV-2, COVID-19, coronavirus, pooling, RT-qPCR, asymptomatic

## Abstract

**Background:** The high COVID-19 dissemination rate demands active surveillance to identify asymptomatic, presymptomatic, and oligosymptomatic (APO) SARS-CoV-2-infected individuals. This is of special importance in communities inhabiting closed or semi-closed institutions such as residential care homes, prisons, neuropsychiatric hospitals, etc., where risk people are in close contact. Thus, a pooling approach—where samples are mixed and tested as single pools—is an attractive strategy to rapidly detect APO-infected in these epidemiological scenarios.

**Materials and Methods:** This study was done at different pandemic periods between May 28 and August 31 2020 in 153 closed or semi-closed institutions in the Province of Buenos Aires (Argentina). We setup pooling strategy in two stages: first a pool-testing followed by selective individual-testing according to pool results. Samples included in negative pools were presumed as negative, while samples from positive pools were re-tested individually for positives identification.

**Results:** Sensitivity in 5-sample or 10-sample pools was adequate since only 2 Ct values were increased with regard to single tests on average. Concordance between 5-sample or 10-sample pools and individual-testing was 100% in the Ct ≤ 36. We tested 4,936 APO clinical samples in 822 pools, requiring 86–50% fewer tests in low-to-moderate prevalence settings compared to individual testing.

**Conclusions:** By this strategy we detected three COVID-19 outbreaks at early stages in these institutions, helping to their containment and increasing the likelihood of saving lives in such places where risk groups are concentrated.

## Introduction

COVID-19, caused by SARS-CoV-2, emerged on December 12, 2019 with 27 cases in Wuhan, China, and spread rapidly, surpassing 45 million infected people and one million deaths all over the world in October 2020. Its symptomatology was classified in six groups that might correlate with illness severity (1, 2). Elderly, and those with underlying medical conditions are at higher risk of developing serious illness. Meanwhile, others can become infected and develop moderate symptoms or even carry the infection asymptomatically. Such asymptomatic, presymptomatic, and oligosymptomatic (APO) people represent a great concern for health system since they may go unnoticed while contributing to SARS-CoV-2 circulation (3–5). In addition, APOs cannot be detected by passive surveillance, which diagnoses only suspicious cases.

Mitigating SARS-CoV-2 circulation necessitates continuous tracking, detection, and isolation of cases, for which active surveillance with massive and opportune APO detection methods is required. A possible strategy may be pooling individual samples for molecular diagnosis. This strategy, which was used successfully for syphilis, HIV, HBV, HCV, *Chlamydia trachomatis* and *Neisseria gonorrhoeae* (6–13), consists of mixing several samples together and then test the pooled samples in one reaction. If the pool test is negative, it may be presumed that all patients are negative, while if it is positive, each sample is separately tested to find out which is responsible for that result. Thus, fewer tests are run overall, saving time and testing supplies, allowing faster return of results in most cases. As expected, when prevalence is low, pooling is usually cost-saving regarding testing samples individually. Using certain algorithms (i.e., dividing positive pools into halves, testing each of the two new smaller pools and continue subdividing positive pools, or 2 two-dimensional array with master pool testing etc.), 60–80% savings were calculated (14–16).

SARS-CoV-2 was isolated and described (17, 18) enabling molecular-diagnosis, which is performed mostly by retrotranscribed quantitative PCR (RT-qPCR) (19). With this technique, the pooling-strategy was assayed with different algorithms, particularly in asymptomatics, since low prevalence is expected there. By implementing the linear eight-sample Dorfman clustering to test 26,576 samples from asymptomatic individuals, 31 (0.12%) SARS-CoV-2 positives were identified, thus achieving a 7.3-fold increase in throughput (20). Moreover, by using a Shiny application (https://www.chrisbilder.com/shiny), efficiency of the pool size was assessed (21).

Special concern exists for SARS-CoV-2 dissemination in closed or semi-closed institutions such as residential care homes, neuropsychiatric hospitals, prison houses, police stations housing prisoners, etc. because they are inhabited by people in close contact that, in addition, have one or more risk factors. If the disease gets access to these vulnerable high-density communities, the demands for hospitalization, complex treatments, and assisted breathing could suddenly increase. To cope with this risk, in this study we implemented an active surveillance through a pooling-strategy aimed at early APOs detection in closed or semi-closed institutions in the Province of Buenos Aires, Argentina (population: 17.5 million, 38.5% of Argentina population) at different moments of the pandemic. The study is part of the active surveillance carried out by the Ministry of Health of the Province of Buenos Aires, and complements the passive surveillance that is being performed from the beginning of the pandemic.

## Materials and Methods

### Sample Collection

Swabs (Britania or any rayon or dacron swab approved by the Argentine regulatory body) from both nostrils and the throat were collected by healthcare providers, and placed immediately into a sterile transport tube containing 2–3 ml of either viral transport medium, Amies transport medium, phosphate buffered saline, or sterile saline. For processing, all samples were properly labeled with the patient's filiation data and accompanied by their corresponding notification forms. Samples thus conditioned were shipped to the VacSal laboratory in refrigerated safety containers, and stored at 2–8°C for a maximum of 3 days, after which they were processed and analyzed.

### RNA Extraction From Individual and Pooled Samples

Sample inactivation and RNA extraction were done using certified class-II biological safety cabinet. RNA was extracted from five-sample and 10-sample pools, as well as from individual samples, using the same RNA extraction kit (RNA Mini Kit Genaid RT300, Geneaid Biotech Ltd) following manufacturer's instructions. Briefly, 200 μl of individual or pooled samples in viral transport media were used for RNA extraction. The individual samples, as well as the pools, were included in the same extraction batch, and the same aliquot was used. Negative pools with 3, 5, or 10 negative samples were included in the assays.

### Retrotranscribed Quantitative PCR (RT-qPCR) for SARS-CoV-2 RdRp, E, ORF1ab, and N Genes

Single-step RT-qPCR for SARS-CoV-2 targeting the RdRp, E and N genes (GeneFinder™ COVID-19 PLUS RealAmp Kit) and ORF1ab and N genes (DisCoVery SARS-CoV-2RT-PCR Detection Kit Rox) was performed on the extracted RNA from individual and pooled samples immediately after RNA extraction.

To assess the sensitivity of the pooling strategy, we arbitrarily chose positive RNA samples with different Ct previously quantified, to prepare *ad hoc* diluted mixes with negative RNA samples. RT-qPCR was performed according to the procedure for individual samples in the clinical laboratory, with identical thermocycler and program (Applied Biosystems® 7500 fast), and with reagents used at the VacSal and Facultad de Ciencias Exactas y Naturales labs. Reaction mixtures using GeneFinder kit were heated to 50°C 20 min for reverse transcription, denatured at 95°C 10 min, and then 50 cycles of amplification were carried out at 95°C 15 s and 58°C 60 s. Fluorescence was measured using the FAM (for RdRp gene), Texas Red (for E gene), JOE (ABI)/VIC (CFX96) (for N gene), and Cy5 (for internal control) channels. Reaction mixtures using DisCoVery kit were heated to 50°C 10 min for reverse transcription, denatured at 95°C 30 s, and then 45 cycles of amplification were carried out at 95°C 5 s and 58°C 34 s. Fluorescence was measured using the FAM (for ORF1ab gene), VIC (CFX96) (for N gene), and Rox (for internal control) channels.

Concordance between individual and pooled sample testings was calculated, and expressed in percentages.

### Determination of the Limit of Detection

The limit of detection for the pooling method was assessed following the protocol already described (22). The test material was RNA obtained from anonymous SARS-CoV-2 negatives and positives, which were collected at Instituto de Investigaciones Biomédicas en Retrovirus y SIDA (INBIRS). First four independent positive RNA extracts with Ct ranging 31–34 were analyzed in 10 replicates of 1:20 pools. Then, one positive RNA sample (Ct 31.7) was analyzed in 20 replicates of different pool sizes (1, 10, 20, 40, 80, and 160). Negative RNAs were used for dilution. RT-qPCRs were performed as described above. The limit of detection for RT-qPCR methods was estimated from analysis of replicate standard curves.

### Surveillance in Semi-closed Institutions

**Figure 1:**
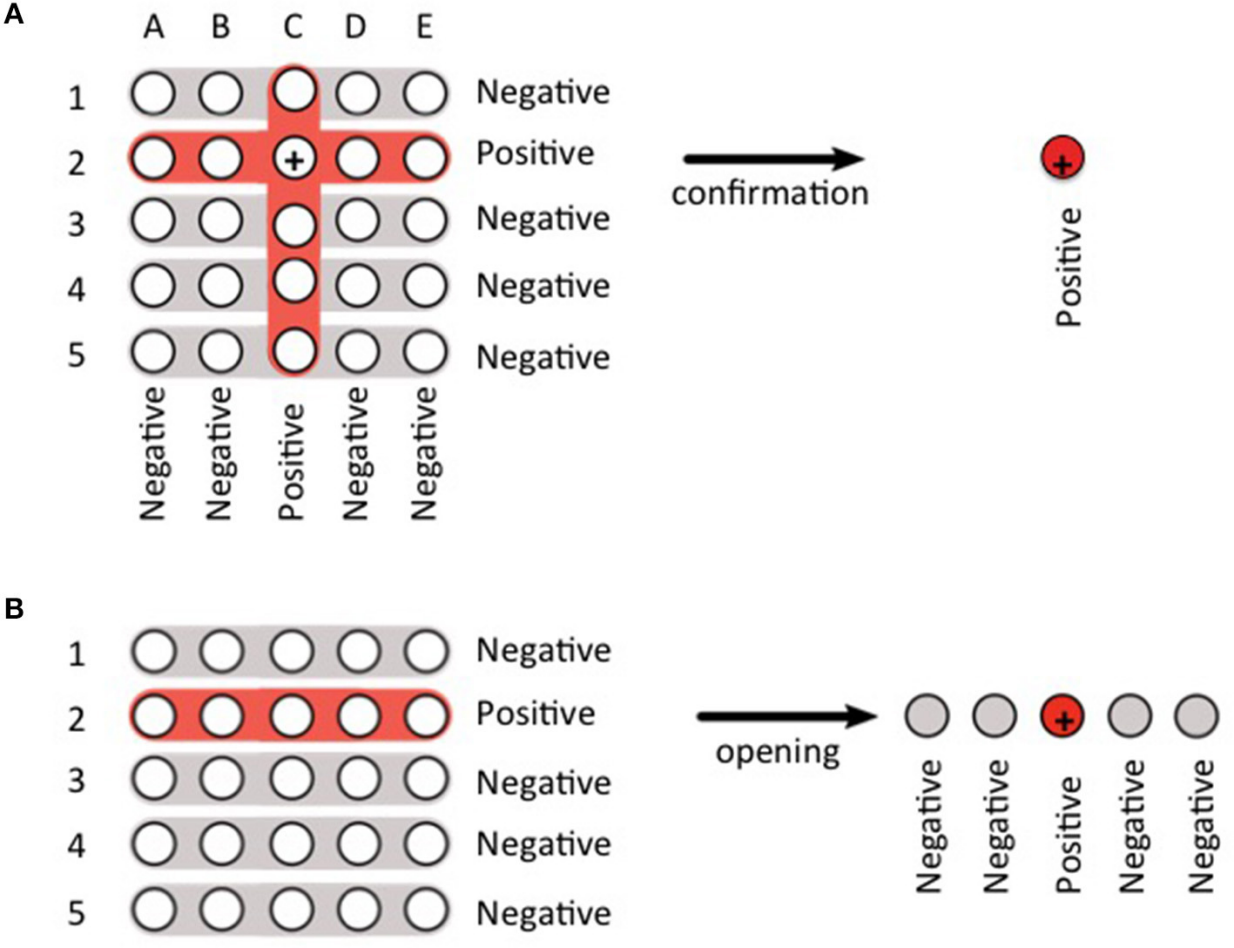
Scheme of the two pooling strategies used in this work. **(A)** square matrix array. Here the samples are grouped in pools 1–5 representing rows, and in pools A–E representing columns. If pools 2 and C are positive, it is concluded that the third sample of pool 2, which is also the second sample of pool C, is positive (+). The result of this sample is confirmed by an individual test. **(B)** linear array. Here the positive pool (in this case, pool 2) must be opened to identify the positive sample (+).

This strategy was implemented since the end of May 2020 as part of surveillance activities coordinated by the Ministry of Health of the Province of Buenos Aires following national and provincial guidance (https://www.argentina.gob.ar/salud/coronavirus-COVID-19/laboratorio, https://www.gba.gob.ar/saludprovincia/noticias/la_provincia_de_buenos_aires_impuls%C3%B3_los_testeos_por_pool_para_evitar_brotes). Informed consent for these diagnostic activities (Public Health activities) is not requested. The VacSal laboratory was chosen to validate and carry out the pool strategy based on the clinical samples obtained from the residents or healthcare workers from closed or semi-closed institutions. At the beginning of the pandemic and when the prevalence of COVID-19 cases was low, sample groupings were performed using a square matrix array (columns and rows). To this end, sample groupings were done in two ways: on the one hand, a group of samples represented a row of the matrix and on the other hand, the samples were grouped again to represent a column of the matrix (Figure 1A). Thus, if only one row and one column tested positive, the positive sample could be identified within the pools (Figure 1A). When the prevalence of COVID-19 cases increased (after June) or when it was unknown, we used the linear grouping array. In this case, if all pools gave negative results in the RT-qPCR, the experimentation was concluded. In contrast, if a positive result was obtained for a pool of samples, then each sample that is included in the pool was tested individually (Figure 1B).

In a 3-month period, 4,936 clinical samples from 153 institutions distributed in 29 municipalities of the Province of Buenos Aires were evaluated.

## Results and Discussion

### Evaluation of Pooling Performance

**Figure 2:**
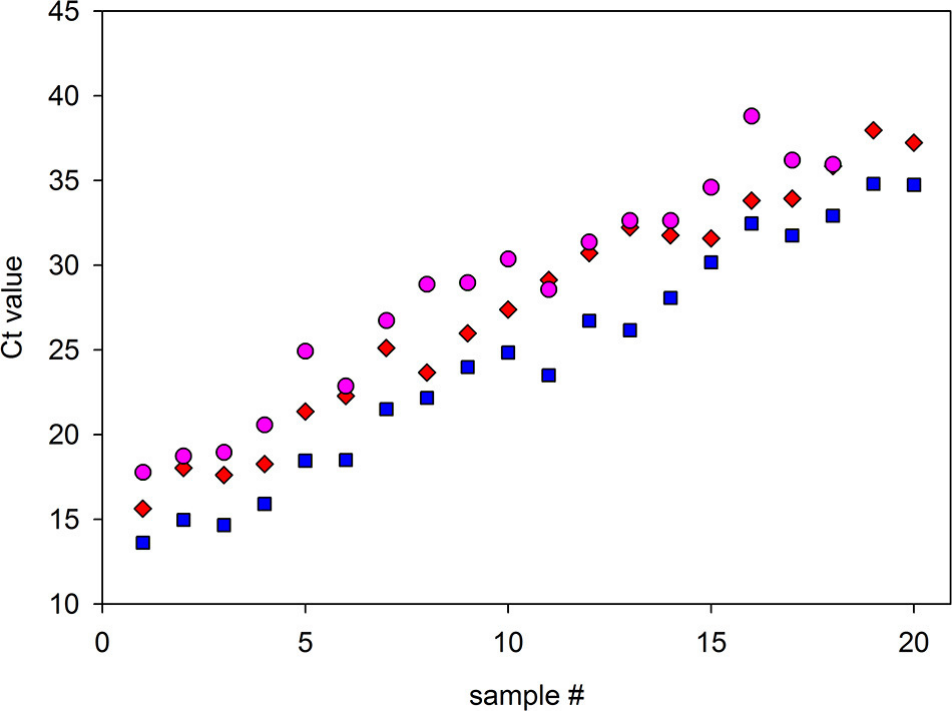
Comparison of RT-qPCR detection of selected samples alone or in pools, and assessment of stability after freezing and thawing. Twenty representative samples with a range of Ct values were subjected to RT-qPCR alone or diluted in 1:20 with negative samples and measured. After dilution, 18 of the 20 samples were detected, while two samples, which possessed the highest Ct values, were not. Red diamonds: Ct values of the samples measured alone (N gene, GeneFinder kit). Pink circles: Ct values of the 1:20 pool. In parallel, Ct values for the same samples measured alone were obtained after freezing and thawing to observe their stability (blue squares). These last two measures were from N gene performed with DisCoVery kit. The average ΔCt value due to the dilution for the measurements at the same run was 4.95, close to the theoretical value (4.3).

From 526 independent anonymous SARS-CoV-2 positive RNAs stored at −70°C for setup studies, 20 representative samples were systematically chosen. Samples were ordered in an equispaced manner by initially measured Ct as depicted in Figure 2, alongside a second measurement of them after being defrozen, and the respective 1:20 pools prepared from original samples.

**Figure 3:**
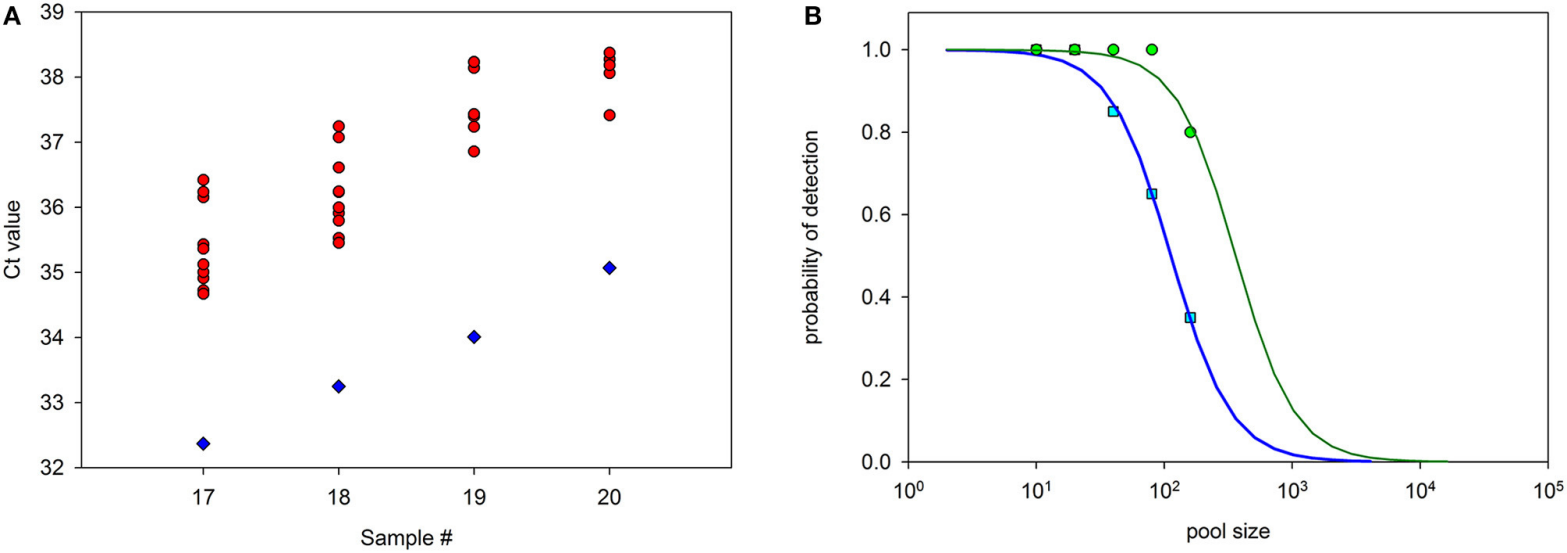
Probability of detection of samples included in pools of different sizes. Samples #17 to #20, which possessed the highest Ct values, were diluted 1:20 with negative samples and measured. **(A)** Diamonds: Original Ct (ORF1) from samples #17 to #20. Circles: Ct values from 10 replicates of the same samples diluted 1:20 with negative samples and measured. Detection with Ct <40 was positive in all dilutions of samples #17 and #18, 7 out of 10 dilutions of sample #19, and 5 out of 10 dilutions of sample #20 (circles with the same Ct value appear superimposed). **(B)** Probability of detection, as determined in 20 replicas as before, for pools containing one positive sample of Ct = 31.7 (ORF1) diluted at 1:10 to 1:160 with negative samples. Blue squares: ORF1 gene. Green circles: N gene. Lines are best fits of sensitivity curves.

To determine the probability of detection near the positive/negative detection boundary, 10 replicates of samples #17 to #20, which possessed the lowest Cts, were analyzed in 1:20 pools. Figure 3A indicates that pools belonging to the samples with original Ct 32.4 and 33.3 were detected at 100% rate, while samples with Ct 34.0 and 35.1 were detected at 70 and 50% rate, respectively. To determine the suitability of the pooling method for a range of dilutions, a study was conducted by comparing 20 replicates of different dilution pools, previously known to be near the edge of the detected/undetected result for ORF1ab and N genes. The result indicated a high rate of detection in pools <1:20 (Figure 3B).

**Figure 4:**
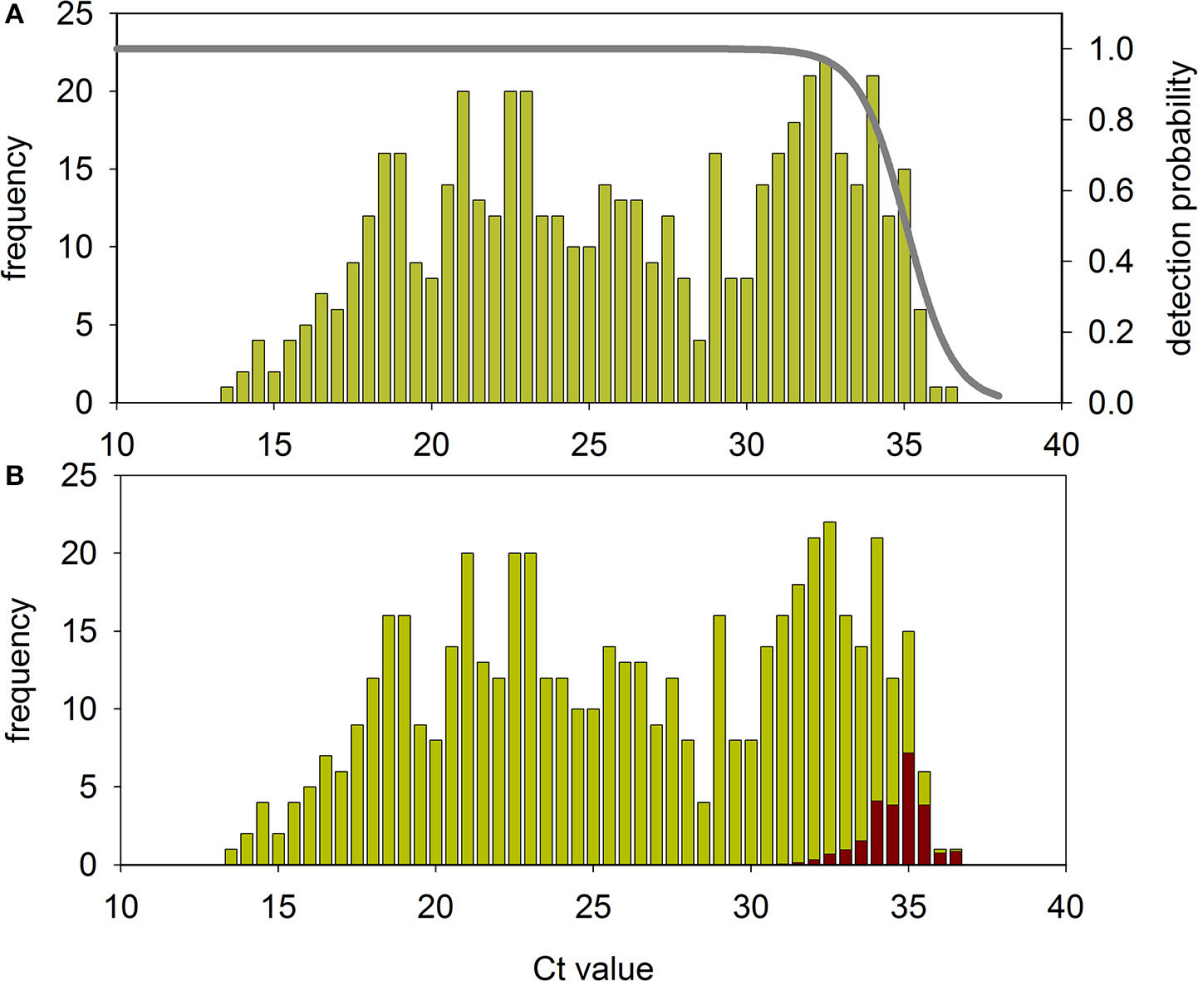
Robustness of the pool method. **(A)** Histogram of the 526 initial diagnostic samples and probability of their individual detection in a 20 samples pool in experiments performed as depicted in Figures 2, 3. **(B)** The same histogram indicating the fraction of samples that will be not detected in a 20 samples pool (red bars).

From the comparison of detection probability and the complete clinical samples Ct histogram, the pool method robustness may be estimated. Figure 4A shows the histogram of all 526 samples ordered by N gene-Ct. Despite samples corresponded to initial diagnostic tests, a bimodal distribution is apparent. This two-peak histogram was as previously reported for SARS-Cov-2 infection (23), although, to our knowledge, no explanation was provided yet. Preliminary results indicate that the bimodality is unrelated with symptoms severity, since asymptomatic individuals also present similar histograms. Cts corresponding to high and low viral loads are rather evenly distributed along samples. The line indicates the probability of positive detection of a single sample in a 20-samples pool. Figure 4B shows detectable and undetectable samples in the histogram. The coincidence value is 95.3%. It is important to note that the 5% that is lost is not evenly distributed among the samples but corresponds to the lowest specimens' viral loads.

### Assessment of Pooling Strategy Sensitivity

The impact of pooling clinical samples aliquots (nasopharyngeal-swabs) prior RNA extraction was tested. To this end, clinical samples with Cts in the ranges of either 20–23 or 30–33 were employed. By combining these samples with negative clinical samples, 1:5 and 1:10 pools were formed. From them, RNA was extracted and RT-qPCR was performed to obtain the Cts. Regardless of individual Ct of the positive RNA extract included in the pool, Cts increased 1.9 ± 1.1 units in 1:5 pools with respect to individual RNA extract, and 2.2 ± 0.3 units in 1:10 pools. Regarding clinical samples whose individual RNA extracts possessed Ct ≥ 36, they turned out negative when pooled together with four or more negative samples.

### Pooling Strategy Applied to Active Surveillance of COVID-19 in Closed or Semi-closed Facilities in the Province of Buenos Aires, Argentina

On the basis of these results and considering that contagiousness of individuals with Ct ≥ 36 would not impact on COVID-19 epidemiology (24–26), the Ministry of Health of the Province of Buenos Aires decided to apply this methodology to analyze health situation in closed or semi-closed facilities.

The first confirmed case in the Province of Buenos Aires was detected on March 8, 5 days later than the first case detected in Argentina. From this date on, the rate of increase in the week average of total number of cases (*N*) as well as in the daily reported cases (*n*) was fast until the end of May, although few cases were still reported. The pooling-strategy was started on May 28, when a significant increase was evident, and data presented here are until August 31. From the slope of plots of log_2_*N* against time, *N* duplication time was deduced for this whole period as 16.1 days; however, *N* duplication time was increasing from 12.4 days between May 28 and July 7 to 29.9 days between August 18 and 31.

From May 28 to August 31, 4,936 samples were received from 153 institutions distributed in 29 municipalities in the Province of Buenos Aires. Between May 28 and July 7 (duplication time 12.4 days) the prevalence of positives in the analyzed samples did not exceed 4% (40 positives out of 1,052 clinical samples analyzed). In these cases, the clinical samples were pooled mostly applying a matrix clustering where samples are arranged in a square matrix with each row and each column tested in a different pool (Figure 1A). Therefore, samples whose row and column are both positive are retested individually. Notice that if there is only one positive sample there will be only one positive row and one positive column. In this scenario the positive sample can be identified at this stage without additional individual testing. So, although this strategy involves more reactions than linear clustering when the number of samples to be tested is small, it allows frequent positives identification without opening pools when prevalence is low. This strategy allowed saving time and kits (66% in average). During a second period comprised between July 8 and August 2 (duplication time 16.9 days) the growth rate in the number of cases was still accelerating for both *n* and *N* values. Hence, given that higher percentage of positivity was expected in this epidemiologic context, the advantage of avoiding opening pools through matrix clustering was lost, so linear clustering (Figure 1B) was implemented. Although at the beginning of this period pools of five samples for RNA extraction and 10 samples (two pools of five) for RT-qPCR were used, during most of the period analyses were done with pools of five clinical samples for both RNA extraction and RT-qPCR. During this second period 1,730 samples were processed, from which 481 were positive (27.8% prevalence). Finally, during August, when duplication time raised to 22.1 days in the first fortnight, and to 29.9 days in the second 1,262 samples were processed, with 358 positives (28.4%) in the first fortnight and 892 samples with 135 positives (15.1%) in the second Thus, the number of people tested per kit (kit saving) ranged from 2.0 to 7.4 depending on the place of origin, with an average of 3.0. Given the high prevalence, this level of savings is quite better than the values predicted by usual mathematical models. This is because pooled samples are not independent of each other since they were obtained from the same closed or semi-closed facility. In other words, since positive samples are unevenly distributed among facilities, pooling-strategy is more effective than predicted.

**Figure 5:**
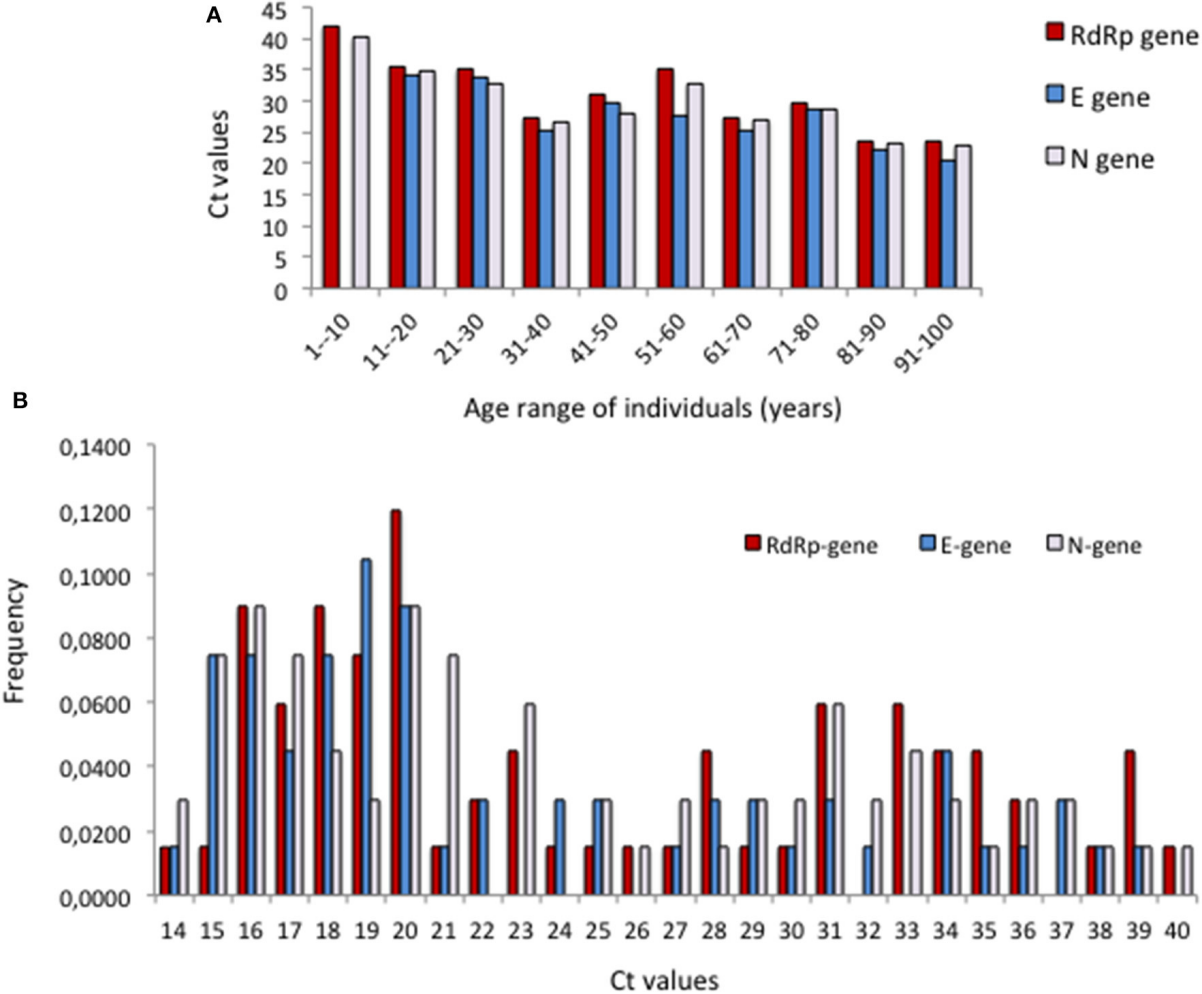
Distribution of Ct values in the clinical samples from asymptomatic individuals. **(A)** Cts according to age for the 10 cohorts between ages 1 and <100 years. **(B)** Frequencies of Cts in the age group 70–100 years.

Because the strategy was used mostly with samples from care homes, the majority were from elderly population. In the group >75-year-old, 30.2% positive cases were detected (1,415 positives in 4,682 clinical samples). Furthermore, there were 44.7% positives in the group 10–14-year-old, and 42.8% in the group 5–9-year-old, albeit from smaller samples (17 positives out of 38 and nine out of 21, respectively). Almost all positive cases were APO, irrespective of age. Interestingly, in asymptomatic cases, the elder group tended to possess lower Ct than younger groups (Figure 5A), being the lowest Cts the most frequent among asymptomatic elders (Figure 5B).

### Early Detection of Infection Foci

Tracing of infection foci and outbreaks was possible in institutions from which samples were received repeatedly. A first example is a women psychiatric hospital at Temperley (Municipality of Lomas de Zamora) from which 216 samples from 210 people distributed in different rooms—including health workers and resident patients—were received and processed on June 19, 23, and 30, July 3, 6, 14, 23, and 28, and August 14 and 20. Analyses were carried out in 106 reactions with a kit saving of 2.6 ± 2.0.

Until June 23 all samples were negative. On June 30 there were five positives, one of which corresponded to an ambulance driver. Therefore, samples from the other drivers and his close family contacts were asked, all of which, as well as a nurse, resulted positive on July 3 (seven positives). Meanwhile, the other samples, which included 17 healthcare workers and 29 residents, were all negative. In view of this situation, isolation procedures were launched and as result, there were no positives 20 days later. However, three new positive cases were detected on July 28, including a nurse. Epidemiologic investigation showed that this nurse has recently been concluded her preventive quarantine because of being close contact of a symptomatic case. Therefore, all patients that were in contact with this nurse were isolated, and analyzed on August 14. It turned out that all 26 patients were positive, and 21 of them developed symptoms. Strict isolation was undertaken, but one of the patients that had comorbidity conditions deceased. Stringent isolation measures were undertaken because focus dissemination to other rooms of the hospital was detected. On the next survey, carried out August 20 among 18 asymptomatic health workers with close contact among them, only 2 were positive.

In another example, a total of 123 samples from 105 different people were received from a disabled center. This center has two sieges, one at Bernal and the other at Quilmes (both in the Municipality of Quilmes). Samples were from young patients, with mean age 24 ± 4 years in Bernal and 23 ± 9 years in Quilmes.

From Bernal, 35 samples from 28 different patients were processed on July 16 and 21, and August 11 (kit saving: 2.0 ± 1.0). On July 16 there were 7 positives out of 15 total samples (47%), while on July 21 all 13 samples analyzed were positive. Therefore, isolation protocols were applied and all negatives from July 16 were re-analyzed. In this second analysis all patients were negative again.

In turn, 88 samples belonging to 77 different patients were received from Quilmes, which were processed in 28 reactions (kit saving: 3.4 ± 1.4). Eleven samples were received twice each; consecutive analyses of these samples resulted either negative in both instances (three cases) or negative in the first analysis and positive in the second (eight cases). RT-qPCRs were carried out at June 30, July 23 and 30, and August 11. On June 30, all results were negative. The first positive was detected on July 23 in a girl that had cardiac antecedents and presented odynophagia, who was negative in the first analysis on June 30. Therefore, samples were obtained from other patients and analyzed on July 30. All these samples, which amounted 37, were positive. At this moment, several of these patients were hospitalized and prevention measures were further stressed. In the new sampling carried out on August 11, positivity was reduced to four positives detected among 11 samples (36%). Three of the negatives observed at August 11 were previously negative on June 30, showing the control of the focus.

## Conclusions

Pooling effectiveness depends on the prevalence of positive samples (27). Therefore, batch sizes for pool testing or even the decision of pool testing should be taken at the laboratory or regional levels, considering positivity rates, specific groups, and categories being tested. Groups with high pre-test probability or serious manifestations are inadequate for pool testing.

Pooling up to 5–10 samples increased test capacity with existing equipment and test kits and detected positives with Cts ≤ 36 with sufficient diagnostic accuracy (2 Cts increase on average). Remarkably, the use of this strategy in the Province of Buenos Aires allowed early outbreaks detection, and evidenced that APOs may present Cts as low as those of symptomatic individuals. The role of APOs in virus transmission must be further studied.

## Data Availability Statement

The original contributions presented in the study are included in the article/supplementary material, further inquiries can be directed to the corresponding author/s.

## Ethics Statement

Ethical review and approval was not required for the study on human participants in accordance with the local legislation and institutional requirements. Written informed consent from the participants' legal guardian/next of kin was not required to participate in this study in accordance with the national legislation and the institutional requirements.

## Author Contributions

NA, PM, KB, DB, MG, VG, AG, ER, and EZ: investigation, writing-review, and editing. RC and FL: resources, investigation, writing-review, and editing. MD, OF, ML, FM, LR-V, and GS: formal analysis, writing-review, and editing. AL: writing-original draft, formal analysis, validation, and visualization. NP: formal analysis, investigation, writing-review, and editing. AP, RE, and DH: conceptualization, methodology, validation, visualization, investigation, resources, writing-original draft, supervision, funding acquisition, and project administration. All authors contributed to the article and approved the submitted version.

## Conflict of Interest

The authors declare that the research was conducted in the absence of any commercial or financial relationships that could be construed as a potential conflict of interest.
